# Disordered Eating Attitudes in Hungarian Adults: Body Image, Sociocultural Media Pressures, and the Cross-Gender Athletic Ideal

**DOI:** 10.3390/nu18152506

**Published:** 2026-08-03

**Authors:** Lina Efthyvoulou, Teodora Dergez, Maria Koushiou, Marios Argyrides

**Affiliations:** 1 Department of Psychology, Neapolis University Pafos, 8042 Paphos, Cyprus; l.efthyvoulou@nup.ac.cy; 2 Faculty of Health Sciences, University of Pécs, 7622 Pécs, Hungary; teodora0925@gmail.com; 3 Department of Social Sciences, University of Nicosia, 2417 Nicosia, Cyprus; koushiou.m@unic.ac.cy

**Keywords:** eating disorders, disordered eating, body image, social media, internalisation, athletic ideal, fitspiration, mental health, nutrition, public health

## Abstract

**Background/Objectives:** Appearance ideals are increasingly transmitted through digital media, reshaping the sociocultural context in which disordered eating develops. Central and Eastern European populations remain underrepresented in this literature. This study examined the prevalence of disordered eating attitudes in a Hungarian adult community sample and tested an integrated model of body image disturbance and media-transmitted sociocultural pressures as statistical predictors, including formal tests of mediation. **Methods:** Hungarian adults (*N* = 675; 70.8% female; aged 16–81 years, *M* = 44.09) completed the Eating Attitudes Test–26 (EAT-26), the Body Attitude Test (BAT), and the Sociocultural Attitudes Towards Appearance Questionnaire–3 (SATAQ-3) in an online survey; body mass index (BMI) was calculated from self-reported height and weight. All instruments demonstrated good to excellent internal consistency in the present sample (Cronbach’s α = 0.84–0.93). **Results:** One in five participants (20.7%) scored at or above the EAT-26 clinical cut-off, with elevated rates at both extremes of the BMI spectrum (underweight: 30.0%; obese: 28.4%). Women scored higher than men on the EAT-26 (*d* = −0.59), BAT (*d* = −0.80), and three SATAQ-3 subscales; no gender difference emerged for athletic-ideal internalisation (*d* = −0.02), consistent with a cross-gender athletic ideal. The predictor set accounted for 40.5% of the variance in EAT-26 scores; BAT was the dominant predictor (β = 0.61), while SATAQ-3 Athletic retained a direct path (β = 0.08), and the remaining media-influence subscales lost significance once body image was controlled. A binary logistic regression using these predictors correctly classified 83.6% of participants (Nagelkerke *R*^2^ = 0.35), with BAT and athletic-ideal internalisation again the only significant predictors of clinical-range classification. Bootstrapped mediation analyses confirmed that the associations between sociocultural influences and disordered eating attitudes were largely mediated by body image disturbance; only athletic-ideal internalisation retained a significant direct association (partial mediation). **Conclusions:** Body image disturbance constitutes the proximal core of disordered eating attitudes, whereas internalisation of the athletic ideal shows a small independent association, with implications for screening and prevention across the adult lifespan.

## 1. Introduction

### 1.1. The Public Health Significance of Disordered Eating

Eating disorders and disordered eating attitudes represent a significant and growing public health concern, associated with substantial psychological distress, medical morbidity, and impaired quality of life [1,2]. While clinical eating disorders such as anorexia nervosa, bulimia nervosa, and binge eating disorder have been extensively studied, a growing body of research has drawn attention to the broader spectrum of disordered eating attitudes that fall below the threshold of formal diagnosis yet carry meaningful psychological and physical consequences [3]. This subclinical range—characterised by distorted eating cognitions, dysfunctional weight-control behaviours, and preoccupation with food and body shape—is considerably more prevalent in the general population than full-threshold disorders and may serve as a risk pathway toward clinical eating pathology [4].

The context in which disordered eating attitudes develop and are maintained has been transformed by the digital media environment. Appearance ideals once transmitted through television, magazines, and interpersonal channels are now communicated continuously through algorithmically curated social media feeds that selectively amplify appearance-focused content in response to user engagement [5]. Meta-analytic and systematic reviews consistently link social media use—particularly appearance-focused use—to body dissatisfaction and disordered eating [6,7], while diet-related misinformation and content normalising restrictive eating circulate online with limited editorial oversight, shaping nutrition attitudes and food choices in community populations [8]. These developments have not redefined the core clinical features of eating pathology, but they have reshaped the sociocultural inputs through which appearance pressures reach individuals—a shift with particular relevance for community samples spanning the adult lifespan, whose current media environments differ markedly from those in which classical sociocultural models and their measures were developed.

The Eating Attitudes Test–26 (EAT-26) [9] is among the most widely used and psychometrically robust instruments for screening disordered eating attitudes across clinical and community populations. A total score at or above the established cut-off of 20 indicates clinically significant disordered eating attitudes warranting further clinical evaluation [9,10]. Studies employing the EAT-26 in non-clinical adult samples have reported prevalence estimates ranging from approximately 10% to 25%, with considerable variation across cultural contexts, age groups, and measurement methodologies [11,12]. Despite this breadth of international evidence, Central and Eastern European populations—including Hungary—remain underrepresented in the eating disorder literature, limiting the generalisability of existing prevalence data and theoretical models to this region [13].

### 1.2. Body Image Disturbance as a Proximal Predictor of Disordered Eating

Among the psychological factors implicated in the aetiology and maintenance of disordered eating, body image disturbance has consistently emerged as one of the most robust and proximal predictors [14,15]. Cognitive-behavioural models of eating pathology, most notably those advanced by Fairburn and colleagues [16], position negative body evaluation as a central maintaining mechanism: distorted or highly negative perceptions of one’s own body generate emotional distress that motivates dietary restraint, compensatory behaviours, and preoccupation with eating and weight. The Body Attitude Test (BAT) [17] was developed to assess the subjective, affective experience of one’s body, extending beyond simple dissatisfaction to capture feelings of bodily alienation, negative body awareness, and a sense of lack of familiarity with one’s own physical self. This broader conceptualisation of body image disturbance renders the BAT particularly sensitive to the cognitive-affective core of eating pathology.

Empirical evidence consistently supports a strong association between BAT scores and measures of disordered eating across diverse samples [17,18]. Cross-cultural studies have further confirmed that body image disturbance, as captured by instruments such as the BAT, retains its predictive relationship with eating attitudes across different national contexts [19], suggesting that its role as a proximal predictor is not limited to Western populations. On this basis, it was hypothesised that BAT scores would emerge as the strongest individual predictor of EAT-26 scores in the present Hungarian sample, in line with established cognitive-behavioural frameworks.

### 1.3. Sociocultural Influences on Body Image and Eating Behaviour in the Digital Era

Sociocultural theories of eating pathology emphasise the role of environmental pressures—transmitted through media, peers, and family—in shaping body image ideals and, by extension, disordered eating attitudes [20,21]. The tripartite influence model [20] proposes that these three sources exert their effects on body dissatisfaction and eating disturbance through two mediating pathways: internalisation of appearance ideals and awareness of appearance-related pressures. In the two decades since the model was formulated, however, the media channel has been radically reconfigured: appearance content is now predominantly encountered through social media platforms, where it is interactive, peer-generated, and algorithmically personalised, blurring the boundary between the media and peer influence pathways of the original model [5,22]. The Sociocultural Attitudes Towards Appearance Questionnaire–3 (SATAQ-3) [23] operationalises the model’s constructs across four subscales: *Information* (awareness of media-communicated appearance norms), *Pressure* (perceived sociocultural pressure to conform to appearance ideals), *Internalisation–General* (endorsement of general thinness and attractiveness ideals), and *Internalisation–Athletic* (endorsement of toned and athletic body ideals). Although the SATAQ-3 was developed before the ascendance of social media—successor instruments such as the SATAQ-4 and SATAQ-4R were subsequently designed to reflect the changed media landscape [24,25]—its Information and Pressure subscales continue to index awareness of, and perceived pressure from, media-communicated appearance norms and thus provide a meaningful, if conservative, window onto media-transmitted influence in the digital era.

Within the tripartite model, Information and Pressure are conceptualised as relatively distal influences—reflecting exposure and perceived demand—whereas Internalisation subscales represent the psychological assimilation of these pressures, positioning them as more proximally linked to body dissatisfaction and eating pathology [23]. Meta-analytic evidence confirms that internalisation of appearance ideals is a stronger predictor of body dissatisfaction and disordered eating than mere awareness or perceived pressure [26]. Importantly, however, when body image disturbance is simultaneously included as a predictor, SATAQ-3 subscales have been shown to lose much of their independent predictive variance [27], consistent with a mediational architecture in which sociocultural pressures exert their effects on eating attitudes largely through their impact on body image. The present study examined whether this pattern would be replicated in a Hungarian adult community sample.

The Internalisation–Athletic subscale merits particular attention in contemporary digital culture. The athletic ideal is the appearance standard most visibly promoted through “fitspiration”—social media content presenting toned, athletic bodies under the banner of health, fitness, and self-discipline [28]. Content analyses indicate that fitspiration material frequently contains objectifying imagery and guilt-inducing messages about weight, dietary restraint, and exercise [29], and experimental exposure to fitspiration imagery has been shown to reduce state body satisfaction and increase negative mood [28]. Unlike thinness ideals, which have historically shown stronger associations with female eating pathology, the digitally amplified athletic ideal is marketed to, and consumed by, both women and men and may therefore represent a cross-gender sociocultural pressure with distinct predictive implications for disordered eating attitudes—potentially operating through pathways not fully captured by body image measures developed in earlier clinical contexts.

### 1.4. Gender Differences in Disordered Eating and Sociocultural Vulnerability

A well-established finding in the eating disorder literature is the substantially greater prevalence of disordered eating attitudes among women compared to men, with female gender consistently identified as one of the strongest demographic risk factors for eating pathology [30,31]. This disparity has been attributed, in part, to the differential sociocultural pressures experienced by women, who are more frequently exposed to and evaluated against narrow appearance ideals communicated through media and social comparison processes [32,33]. Women typically report higher levels of body dissatisfaction, greater internalisation of appearance ideals, and more pronounced sociocultural pressure than men, all of which are theoretically linked to elevated disordered eating risk [15].

Nevertheless, the gender gap in eating pathology has shown signs of narrowing in recent decades, particularly among younger cohorts and in contexts where athletic and muscularity ideals have gained cultural prominence [34]. Men are increasingly subject to body-related sociocultural pressures oriented around fitness, leanness, and muscularity [35], and male eating pathology—including drive for thinness, dietary restraint, and muscle dysmorphia—may be inadequately captured by instruments historically validated in female samples. Of particular relevance to the present study is the prediction that gender differences on the SATAQ-3 Athletic subscale may be attenuated or absent relative to other subscales, given the convergence of fitness-oriented appearance ideals across genders in contemporary culture [28].

### 1.5. BMI, Body Weight, and the Complexity of the Weight–Eating Pathology Relationship

The relationship between BMI and disordered eating attitudes is complex and non-linear. While elevated BMI has been associated with increased body dissatisfaction and binge eating behaviours, particularly in the overweight and obese range [36], low BMI has been linked to restrictive eating cognitions and compensatory behaviours consistent with anorexic symptomatology [9]. Research employing community samples has documented elevated rates of disordered eating at both ends of the BMI spectrum, suggesting a potential bimodal or U-shaped association [37]. This pattern has important implications for screening: weight-based identification of at-risk individuals risks overlooking disordered eating in normal-weight individuals who nonetheless endorse significant eating-related psychopathology while simultaneously failing to differentiate clinically meaningful distress at either weight extreme. The inclusion of BMI as a covariate in the present regression analyses was intended to account for this confound and to examine its unique contribution to disordered eating attitudes when psychological and sociocultural factors are simultaneously controlled.

### 1.6. The Present Study

The present study examined disordered eating attitudes and their psychological and sociocultural correlates in a community sample of Hungarian adults. Despite the considerable international literature on eating disorder risk factors, integrated models incorporating body image disturbance (BAT), sociocultural appearance pressures (SATAQ-3), and demographic variables have not been systematically tested in Hungarian adult community samples. Hungary represents an informative cultural context: as a Central European nation with a distinct historical relationship to body ideals, gender norms, and media culture, it offers an opportunity to examine the cross-cultural generalisability of eating disorder models developed primarily in Western populations. In doing so, the study speaks to a question of growing salience in the digital era: how media-transmitted appearance pressures—now overwhelmingly digital in origin—relate to body image disturbance and disordered eating attitudes across the adult lifespan, in a cultural context underrepresented in the international literature.

Three hypotheses guided the investigation. Hypothesis 1 (H1) proposed that a clinically significant proportion of the Hungarian adult sample would score at or above the EAT-26 cut-off score of 20, indicating the presence of disordered eating attitudes in a meaningful segment of the community population. Hypothesis 2 (H2) proposed that significant gender differences would be observed across disordered eating attitudes and associated constructs, with female participants expected to report higher scores on the EAT-26, BAT, and most SATAQ-3 subscales; it was further expected that the SATAQ-3 Athletic subscale would show a smaller or non-significant gender difference given the cross-gender convergence of athletic ideals. Hypothesis 3 (H3) proposed that BAT and SATAQ-3 subscale scores, along with BMI, would collectively account for significant variance in EAT-26 scores, with BAT expected to emerge as the dominant predictor. It was further anticipated that SATAQ-3 subscales would lose independent predictive variance once BAT was controlled, consistent with a mediational model in which sociocultural pressures operate on eating attitudes primarily through their effect on body image disturbance. This mediational structure was tested formally using bootstrapped indirect-effect analyses.

## 2. Materials and Methods

### 2.1. Participants

The sample consisted of 675 Hungarian adults recruited through an online survey platform. Participants ranged in age from 16 to 81 years (*M* = 44.09, *SD* = 13.69). The majority of participants were female (*n* = 478, 70.8%), while 197 (29.2%) identified as male. In terms of educational attainment, the largest proportion held a secondary school qualification (31.1%), followed by a bachelor’s degree (29.5%), a master’s degree (19.3%), a vocational qualification (14.4%), primary school education (3.1%), a doctoral degree (1.5%), or other qualifications (1.2%). Regarding employment status, 55.7% were employed full-time, 12.3% were self-employed, 10.1% were retired, 8.1% were students, 4.4% were employed part-time, 3.9% were on parental leave, 2.4% were unemployed, and 3.1% reported other employment statuses. The majority of participants were married (49.8%), followed by those in a relationship (21.2%), single (19.7%), divorced (5.9%), widowed (2.2%), and other (1.2%). Descriptive statistics for continuous demographic and anthropometric variables are presented in Table 1, overall and separately by gender; men and women did not differ significantly in age or BMI.

### 2.2. Measures

The three instruments were selected on the following grounds: (a) the EAT-26, BAT, and SATAQ-3 are among the most widely used measures of their respective constructs internationally, maximising comparability with the cross-cultural literature; (b) validated Hungarian adaptations are available for the BAT [38], and the EAT has an established history of use in Hungarian populations, dating to early prevalence work in university students [39]; (c) the SATAQ-3 directly operationalises the constructs of the tripartite influence model that framed the study’s hypotheses; and (d) the combined battery is brief enough for reliable online administration in a community sample. The SATAQ-3 was retained in preference to its successors (SATAQ-4/4R) because it permits direct comparison with the existing cross-cultural literature employing the same instrument; the implications of this choice are considered in the Limitations. Internal consistency coefficients obtained in the present sample are reported below for each instrument.

#### 2.2.1. Eating Attitudes Test–26 (EAT-26)

Disordered eating attitudes were assessed using the Eating Attitudes Test–26 (EAT-26) [9], a widely used self-report instrument measuring the presence of eating-related psychopathology. Items are rated on a six-point Likert scale, with higher scores indicating greater severity of disordered eating cognitions and behaviours. A total score of 20 or above is considered the clinical cut-off indicative of significant disordered eating attitudes [9]. For the purposes of inferential analyses, a dichotomous variable was derived from the continuous EAT-26 score, classifying participants as either “no disordered eating” (score < 20) or “disordered eating” (score ≥ 20).

#### 2.2.2. Body Attitude Test (BAT)

Body image disturbance was measured using the Body Attitude Test (BAT) [17], a 20-item self-report questionnaire assessing the subjective experience of one’s body, including negative appreciation of body size, lack of familiarity with one’s own body, and general body dissatisfaction. Items are rated on a six-point scale (0 = never to 5 = always), yielding a total score between 0 and 100, with higher scores reflecting greater body image disturbance. The validated Hungarian adaptation of the BAT was used [38]. In the present sample, internal consistency was excellent (Cronbach’s α = 0.93).

#### 2.2.3. Sociocultural Attitudes Towards Appearance Questionnaire–3 (SATAQ-3)

Sociocultural influences on body image and eating behaviour were assessed using the SATAQ-3 [23], which comprises four subscales: *Information* (awareness of media-communicated appearance ideals), *Pressure* (perceived sociocultural pressure to conform to appearance standards), *Internalisation–General* (internalisation of general appearance ideals), and *Internalisation–Athletic* (internalisation of athletic body ideals). Higher scores on each subscale reflect greater endorsement of the respective sociocultural construct. In the present sample, internal consistency was good to excellent for all subscales (Information: α = 0.89; Pressure: α = 0.91; Internalisation–General: α = 0.93; Internalisation–Athletic: α = 0.86).

### 2.3. Research Hypotheses

The present study was guided by three research hypotheses. Hypothesis 1 (H1) proposed that a clinically significant proportion of the Hungarian adult sample would score at or above the EAT-26 cut-off score of 20, indicating the presence of disordered eating attitudes. Hypothesis 2 (H2) proposed that significant gender differences would be observed in disordered eating attitudes and associated psychological constructs, with female participants expected to report higher scores on the EAT-26, BAT, and SATAQ-3 subscales compared to male participants. Hypothesis 3 (H3) proposed that BAT and SATAQ-3 subscale scores, along with BMI, would collectively and individually account for significant variance in EAT-26 scores, with body image disturbance (BAT) expected to emerge as the strongest predictor of disordered eating attitudes. It was further hypothesised that the associations between the SATAQ-3 subscales and EAT-26 scores would be mediated by body image disturbance; this mediational structure was tested formally using bootstrapped indirect-effect analyses.

### 2.4. Procedure

The study protocol received approval from the NUP Ethics Committee (Protocol No. NUP 169/2025), and every stage of the research complied with the Declaration of Helsinki [40]. Between September and December 2025, Hungarian adults completed an anonymous self-report questionnaire hosted on Google Forms, with all instruments presented in Hungarian. Before accessing the survey, participants read a description of the study’s purpose, the voluntary basis of taking part, the confidential handling of their responses, and their freedom to discontinue at any point without penalty and then indicated their consent electronically. The questionnaire, which took roughly 10–15 min to complete, consisted of demographic items followed by the three psychometric measures described above; height and weight were self-reported and used to compute BMI.

### 2.5. Data Analysis

All analyses were conducted using IBM SPSS Statistics (Version 29), with mediation analyses and supplementary diagnostics conducted in Python 3.12 (statsmodels). Internal consistency was estimated with Cronbach’s α for each instrument. Descriptive statistics (means, standard deviations, frequencies, and percentages) were computed to characterise the sample and address H1. To test H2, independent samples *t*-tests were conducted to compare male and female participants on EAT-26, BAT, and SATAQ-3 subscale scores. Where Levene’s test indicated violation of the assumption of homogeneity of variance (*p* < 0.05), Welch’s corrected degrees of freedom were reported. Effect sizes were estimated using Cohen’s *d*, interpreted according to conventional benchmarks (small: 0.20–0.49; medium: 0.50–0.79; large: ≥0.80) [41]. The association between BMI category and disordered eating classification was examined using Pearson’s chi-square test. Gender differences in age and BMI were examined in the same manner as the psychometric variables. Associations between age and the study variables were examined with Pearson correlations, and differences in disordered eating across age groups (16–29, 30–44, 45–59, and 60+ years) were tested with one-way ANOVA and chi-square tests. To test H3, a simultaneous multiple linear regression was conducted with EAT-26 total score as the dependent variable and BMI, BAT, and the four SATAQ-3 subscales as predictors. Multicollinearity was assessed with variance inflation factors (VIFs), and the linear model was re-estimated with age as an additional predictor. To formally test the hypothesised mediational structure, four mediation models were estimated (one per SATAQ-3 subscale as the independent variable), with BAT scores as the mediator, EAT-26 scores as the outcome, and BMI as a covariate, using ordinary least squares path analysis with percentile bootstrapped confidence intervals for the indirect effect (5000 resamples), consistent with the approach implemented in the PROCESS macro (Model 4) [42]. A binary logistic regression was subsequently conducted examining how well BAT and the four SATAQ-3 subscales classified participants into disordered-eating (EAT-26 ≥ 20) versus non-disordered-eating (EAT-26 < 20) groups.

## 3. Results

### 3.1. Prevalence of Disordered Eating Attitudes (H1)

The distribution of EAT-26 scores indicated that 140 participants (20.7%) met or exceeded the clinical cut-off score of 20, consistent with the presence of significant disordered eating attitudes, while the remaining 535 participants (79.3%) scored below this threshold. These findings support H1, demonstrating that a clinically meaningful proportion of the Hungarian adult sample reported levels of disordered eating attitudes warranting clinical attention.

Disordered eating attitudes declined significantly with age. EAT-26 scores correlated negatively with age (*r* = −0.23, *p* < 0.001), and the proportion of participants at or above the clinical cut-off decreased monotonically across age groups: 34.0% among participants aged 16–29 years, 25.8% at 30–44 years, 15.3% at 45–59 years, and 8.8% among those aged 60 years and above, χ^2^(3) = 26.63, *p* < 0.001. A parallel gradient was observed for body image disturbance (BAT: *r* = −0.24, *p* < 0.001; age-group means of 41.72, 40.03, 32.01, and 27.48, respectively, *F*(3, 665) = 15.67, *p* < 0.001). Thus, although disordered eating attitudes were present in all age strata—including nearly one in eleven adults aged 60 and above—both eating pathology and body image disturbance were the most pronounced among younger adults.

The relationship between BMI category and disordered eating classification was examined using Pearson’s chi-square test. Results indicated a statistically significant association between BMI category and disordered eating group membership, χ^2^(3) = 10.09, *p* = 0.018. Inspection of the cell frequencies (see Table 2) revealed that disordered eating was the most prevalent among participants classified as underweight (30.0%) and obese (28.4%), relative to those with healthy weight (18.4%) or overweight status (16.5%). It should be noted that one cell had an expected count below 5 (minimum expected count = 4.11), which warrants cautious interpretation of the chi-square statistic.

### 3.2. Gender Differences in Disordered Eating and Related Constructs (H2)

Independent samples *t*-tests were conducted to compare male (*n* = 197) and female (*n* = 478) participants on EAT-26 scores, BAT scores, and all four SATAQ-3 subscales. Results are presented in Table 3. Given significant violations of the homogeneity of variance assumption for several variables (SATAQ-3 Pressure, SATAQ-3 Internalisation, BAT, and EAT-26), Welch’s *t*-test with corrected degrees of freedom is reported for those variables.

Female participants scored significantly higher than male participants on EAT-26 total scores (*M* = 13.69 vs. *M* = 8.24), *t*(456.66) = −7.71, *p* < 0.001, Cohen’s *d* = −0.59, indicating a medium effect. Female participants also reported significantly higher body image disturbance on the BAT (*M* = 40.07 vs. *M* = 25.16), *t*(550.44) = −11.18, *p* < 0.001, Cohen’s *d* = −0.80, representing a large effect size. Significant gender differences were observed across three of the four SATAQ-3 subscales: SATAQ-3 Information, *t*(382.67) = −3.39, *p* < 0.001, *d* = −0.28; SATAQ-3 Pressure, *t*(415.14) = −4.54, *p* < 0.001, *d* = −0.36; and SATAQ-3 Internalisation, *t*(458.51) = −5.03, *p* < 0.001, *d* = −0.39, all reflecting small-to-medium effects. No significant gender difference was found for SATAQ-3 Athletic, *t*(382.56) = −0.19, *p* = 0.847, *d* = −0.02. These results partially support H2: significant gender differences were observed for eating attitudes, body image disturbance, and most sociocultural pressure subscales, though internalisation of athletic ideals did not differ between genders.

### 3.3. Predictors of Disordered Eating Attitudes (H3)

#### 3.3.1. Multiple Linear Regression

A simultaneous multiple linear regression was conducted to examine the extent to which BMI, BAT scores, and the four SATAQ-3 subscales predicted continuous EAT-26 scores. The overall model was statistically significant, *F*(6, 664) = 75.40, *p* < 0.001, *R*^2^ = 0.41, indicating that the predictor set accounted for approximately 40.5% of the variance in disordered eating attitudes (adjusted *R*^2^ = 0.40). Regression coefficients are presented in Table 4. Variance inflation factors indicated no problematic multicollinearity among the predictors (all VIFs ≤ 3.78).

BAT emerged as the strongest individual predictor of EAT-26 scores (β = 0.61, *p* < 0.001), confirming that body image disturbance accounted for the largest unique contribution to disordered eating attitudes. SATAQ-3 Athletic also significantly predicted EAT-26 scores (β = 0.08, *p* = 0.037), albeit with a substantially smaller standardised coefficient. BMI was a significant negative predictor (β = −0.08, *p* = 0.014), suggesting that when body image disturbance and sociocultural factors are controlled, higher BMI was associated with marginally lower disordered eating attitude scores. The remaining SATAQ-3 subscales (Information, Pressure, and Internalisation) did not contribute significantly to the model (all *p* > 0.10). These findings provide support for H3, with BAT confirmed as the dominant predictor of disordered eating attitudes.

Two follow-up analyses were conducted. First, to examine the role of age, the model was re-estimated with age included as an additional predictor. Age was not independently associated with EAT-26 scores (β = −0.05, *p* = 0.133), and the overall pattern of results was unchanged (*R*^2^ = 0.409), although the coefficient for SATAQ-3 Athletic was attenuated to marginal significance (β = 0.07, *p* = 0.065), reflecting the negative association between age and athletic-ideal internalisation (*r* = −0.30, *p* < 0.001). Second, the negative BMI coefficient was examined directly for suppression [43]. At the bivariate level, BMI was positively associated with EAT-26 scores (*r* = 0.16, *p* < 0.001) and with BAT scores (*r* = 0.41, *p* < 0.001); however, when BAT was entered alongside BMI, the BMI coefficient reversed sign (*B* = 0.25, *p* < 0.001 alone vs. *B* = −0.17, *p* = 0.001 with BAT controlled). This sign reversal constitutes a classic suppression pattern [43], indicating that the positive bivariate association between BMI and disordered eating attitudes is carried by body image disturbance.

#### 3.3.2. Binary Logistic Regression

A binary logistic regression was conducted to assess the ability of the BAT and SATAQ-3 subscales to classify participants into disordered eating (EAT-26 ≥ 20) versus non-disordered eating (EAT-26 < 20) groups. The overall model was statistically significant, χ^2^(5) = 172.05, *p* < 0.001, Nagelkerke *R*^2^ = 0.35. The model correctly classified 83.6% of participants overall (94.6% of the non-disordered eating group; 41.4% of the disordered eating group). Logistic regression coefficients are presented in Table 5.

Consistent with the linear regression, BAT was the only variable achieving significance as a predictor of disordered eating group membership (Wald = 90.86, *p* < 0.001, Exp(*B*) = 1.07), indicating that each one-unit increase in BAT score increased the odds of belonging to the disordered eating group by approximately 6.5%. SATAQ-3 Athletic also emerged as a significant predictor (Wald = 5.50, *p* = 0.019, Exp(*B*) = 1.07), suggesting that internalisation of athletic body ideals contributed incrementally to the probability of disordered eating classification, over and above body image disturbance. The remaining SATAQ-3 subscales (Information, Pressure, and Internalisation–General) did not reach significance (all *p* > 0.30). Taken together, these findings corroborate the linear regression results and provide further support for H3.

### 3.4. Mediation Analyses

To test the hypothesis that sociocultural appearance pressures relate to disordered eating attitudes through body image disturbance, four mediation models were estimated, each with one SATAQ-3 subscale as the independent variable, BAT total score as the mediator, EAT-26 total score as the outcome, and BMI as a covariate; indirect effects were tested with 5000 percentile bootstrap resamples (Table 6). For the Information, Pressure, and Internalisation–General subscales, the direct effects on EAT-26 scores were non-significant once body image disturbance was controlled (all *p* ≥ 0.056), while the indirect effects through the BAT were significant, indicating full (or near-full) mediation—the indirect path accounted for 96.5%, 79.6%, and 81.8% of the total effect, respectively. For the Internalisation–Athletic subscale, both the indirect effect (*B* = 0.42, 95% CI [0.32, 0.52]) and a smaller but significant direct effect (*B* = 0.17, *p* = 0.004) were observed, indicating partial mediation, with approximately 71% of the total effect carried through body image disturbance. These results support the hypothesised mediational structure and are consistent with the divergence between the bivariate correlations and the multiple regression reported above.

## 4. Discussion

The present study investigated the prevalence of disordered eating attitudes, gender differences in eating-related constructs, and the psychological and sociocultural predictors of disordered eating in a community sample of 675 Hungarian adults. Three hypotheses were tested. H1 was fully supported: 20.7% of the sample scored at or above the EAT-26 clinical cut-off, confirming that a clinically meaningful proportion of the community population endorsed significant disordered eating attitudes. H2 was partially supported: robust gender differences were found for EAT-26, BAT, and three of the four SATAQ-3 subscales, with the SATAQ-3 Athletic subscale representing a notable exception in which no gender difference was observed. H3 was fully supported: the predictor set accounted for 40.5% of the variance in EAT-26 scores, with BAT emerging as the overwhelmingly dominant predictor; the SATAQ-3 Athletic subscale retained independent predictive significance, while the remaining SATAQ-3 subscales did not. Formal mediation analyses confirmed the hypothesised structure: the associations of all four SATAQ-3 subscales with disordered eating attitudes were mediated by body image disturbance—fully so for Information, Pressure, and Internalisation–General and partially for Internalisation–Athletic, which retained a significant direct association. In addition, disordered eating attitudes and body image disturbance declined with age, although age carried no independent association with eating attitudes once the psychological predictors were controlled.

### 4.1. Prevalence of Disordered Eating Attitudes in Hungary

The finding that 20.7% of Hungarian community adults scored at or above the EAT-26 cut-off of 20 is notable and clinically significant. This figure situates the Hungarian sample toward the upper range of prevalence estimates reported in comparable community studies internationally, which have typically reported rates of 10–25% depending on sample composition, measurement approach, and cultural context [11,12]. The result is consistent with the hypothesis that disordered eating attitudes are not confined to clinical or high-risk populations but are present at clinically meaningful levels across the general adult population. From a public health perspective, these findings argue for the integration of routine eating attitude screening within primary care and occupational health settings in Hungary, particularly given that the mean age of the sample was 44 years, indicating that disordered eating attitudes are not exclusively a concern of adolescence or young adulthood.

A Hungarian benchmark places this figure in sharper relief. In the only Hungarian study to have examined temporal change in disordered eating using the same instrument, the proportion of secondary school girls scoring above the EAT-26 threshold doubled over a decade, from 5.0% in 1989 to 10.2% in 1998/1999, with a corresponding rise in the prevalence of full and subclinical eating disorder syndromes from 0.12% to 0.68%; among boys, above-threshold scores rose from 0% to 0.8% [44]. The authors attributed this increase to the growing cultural salience of physical appearance and the media-transmitted thinness ideal [44]. Against that baseline, the rates observed here roughly two and a half decades later are substantially higher: 25.3% of women and 9.6% of men in the present adult sample scored at or above the clinical cut-off. Such a comparison must be made cautiously, since the samples differ in age range, sampling frame, and mode of administration, and the present study recruited a self-selected online community sample rather than a school-based one. Nevertheless, the direction is consistent with the upward trajectory documented in the Hungarian adolescent literature and suggests that the phenomenon has not remained confined to younger cohorts.

The age gradient observed in the present sample refines this conclusion. Disordered eating attitudes were most prevalent among the youngest adults (34.0% at 16–29 years) and declined progressively to 8.8% among adults aged 60 years and above, with a parallel decline in body image disturbance. This pattern is consistent with international evidence that eating pathology peaks in adolescence and early adulthood [3,31], yet the persistence of clinically elevated scores in nearly one in eleven older adults cautions against treating disordered eating as exclusively a young person’s concern. Screening across the adult lifespan therefore remains warranted, with heightened vigilance in younger cohorts.

The analysis of the relationship between BMI category and disordered eating group membership revealed a theoretically compelling pattern. Disordered eating was most prevalent at both ends of the weight spectrum: among underweight participants (30.0%) and obese participants (28.4%), with comparatively lower rates among those with healthy weight (18.4%) or overweight status (16.5%). This non-linear, bimodal distribution is consistent with a dual-pathway model in which disordered eating attitudes at lower BMI are driven primarily by restrictive and compensatory cognitions characteristic of anorexic-spectrum pathology, while elevated rates at higher BMI reflect binge eating-related distress, emotional eating, and the profound body dissatisfaction that frequently accompanies obesity [36,37]. This pattern carries important clinical implications: screening approaches that rely on weight status as a proxy for eating disorder risk will systematically miss disordered eating in normal-weight individuals and will fail to differentiate the qualitatively distinct presentations at the underweight and obese poles. The present findings underscore the necessity of psychometric screening independent of BMI. It should be noted that the chi-square analysis was limited by a cell count below the recommended threshold of 5 in the underweight/disordered eating cell (expected count = 4.11), and these findings should be interpreted with appropriate caution and replicated in larger samples.

### 4.2. Gender Differences in Disordered Eating and Associated Constructs

The gender comparisons yielded a pattern of results that both confirmed established findings and contributed a theoretically meaningful departure. As expected, female participants reported significantly higher scores on the EAT-26 (*d* = −0.59) and BAT (*d* = −0.80) than male participants, consistent with the robust literature documenting female vulnerability to eating pathology and body image disturbance [15,30]. The large effect size observed for BAT is particularly striking and suggests that the gender difference in body image disturbance is not merely a statistical artefact but reflects a substantively different relationship between women and the subjective experience of their bodies—a difference that is likely rooted in the greater objectification, appearance scrutiny, and sociocultural pressure directed at women across the life course [32].

Significant gender differences were also found for three SATAQ-3 subscales—Information (*d* = −0.28), Pressure (*d* = −0.36), and Internalisation–General (*d* = −0.39)—indicating that women in the present sample were more exposed to, pressured by, and internalising of general appearance ideals than men. Although effect sizes were in the small-to-medium range, the consistency of these findings across subscales is theoretically coherent: women are disproportionately targeted by appearance-related media content and social comparison pressures, and the tripartite influence model would predict precisely this pattern of elevated sociocultural exposure translating into heightened internalisation [20].

The most theoretically significant finding within H2 was the absence of any gender difference on the SATAQ-3 Athletic subscale (*d* = −0.02, *p* = 0.847). This null finding was anticipated in the hypothesis but is nonetheless noteworthy in the context of the broader results. While women showed substantially higher internalisation of general appearance ideals, the endorsement of athletic and toned body standards was statistically indistinguishable between men and women in this Hungarian adult sample. This result is consistent with the argument that contemporary fitness culture has generated a cross-gender athletic ideal that operates as a shared sociocultural standard for both sexes [28,29]. For men, the athletic ideal may articulate with a drive for muscularity rather than thinness, while for women it may co-exist with thinness ideals or represent a culturally sanctioned reframing of body control aspirations. The convergence of athletic internalisation across genders in this sample may partly explain why the SATAQ-3 Athletic subscale retained unique predictive variance in the regression models, despite the other SATAQ-3 subscales losing significance—a point discussed in detail below.

### 4.3. Predictors of Disordered Eating: The Primacy of Body Image

The regression analyses provided strong and consistent support for H3. In the multiple linear regression, the predictor set accounted for 40.5% of the variance in EAT-26 scores—a substantial proportion for a community sample using self-report psychometrics. BAT was overwhelmingly the dominant predictor (β = 0.61, *p* < 0.001), a standardised coefficient of exceptional magnitude that reflects the tight coupling between subjective body image disturbance and disordered eating cognitions in this sample. This finding is entirely consistent with cognitive-behavioural models of eating pathology, which position negative body evaluation as the central maintaining mechanism of disordered eating: when individuals experience their own bodies as alien, repulsive, or fundamentally unacceptable, the cognitive and behavioural sequelae—dietary restriction, compensatory behaviour, preoccupation with food and weight—follow with a high degree of regularity [16]. The replication of this finding in a Hungarian community sample strengthens the claim that BAT taps the psychological core of disordered eating across cultural contexts.

The pattern of results for the SATAQ-3 subscales deserves close interpretive attention. In the independent samples *t*-tests, all four SATAQ-3 subscales significantly differentiated the disordered eating from the non-disordered eating group, with medium effect sizes for Internalisation–General (*d* = −0.72) and Internalisation–Athletic (*d* = −0.60). Yet in the regression model—where BAT was simultaneously included—only SATAQ-3 Athletic retained significant independent predictive variance (β = 0.08, *p* = 0.037). The remaining three subscales (Information, Pressure, Internalisation–General) dropped to non-significance (all *p* > 0.10). This divergence between bivariate and multivariate results was confirmed by the formal mediation analyses (Section 3.4): the associations of Information, Pressure, and Internalisation–General with disordered eating attitudes were fully (or almost fully) mediated by body image disturbance, consistent with a structure in which sociocultural pressures relate to eating attitudes primarily through their association with body image. Sociocultural exposure and internalisation may be necessary antecedents of body image disturbance, but once the latter is statistically controlled, they contribute little additional explained variance. This finding aligns with evidence from mediation studies using the tripartite influence framework, which consistently demonstrate that body dissatisfaction mediates the relationship between sociocultural pressures and eating pathology [26,27]. It must be emphasised, however, that these are cross-sectional mediation models: they establish that the data are consistent with the hypothesised ordering of constructs, not that this ordering is causal. Longitudinal and experimental designs remain necessary to establish temporal precedence.

The exception to this pattern—the SATAQ-3 Athletic subscale—is the most theoretically novel finding of the present study, and the one that connects most directly to the contemporary digital appearance environment. Athletic internalisation retained a small but significant direct predictive path to EAT-26 scores in both the linear regression (β = 0.08) and the logistic regression (Exp(*B*) = 1.07), even after BAT was controlled, and was the only sociocultural construct on which men and women did not differ. Both findings are consistent with—although, in the absence of direct social media measures, cannot confirm—the character of the athletic ideal as it is currently propagated: fitspiration content is among the most pervasive appearance-related genres on social media, is produced and consumed by both genders, and packages body surveillance and dietary control in the socially sanctioned language of health and discipline [28,29]. Because engagement-driven recommendation algorithms amplify such content for users who interact with fitness and nutrition material [5], individuals who internalise the athletic ideal are likely to inhabit self-reinforcing digital environments in which that ideal is continuously restated. One plausible explanation for the residual direct path is that the BAT-16, developed in clinical populations characterised primarily by weight- and shape-related preoccupation associated with thinness norms [17], may not fully capture the body-related distress arising from athletic-ideal internalisation — concerns about muscle tone, leanness, physical performance, and disciplined control over the body that are phenomenologically distinct from simple weight and shape dissatisfaction. The athletic internalisation pathway may thus represent a partially independent cognitive route to disordered eating — plausibly linked to orthorexic tendencies, compulsive exercise, and the pursuit of a “lean” physique — that contemporary digital fitness culture is well positioned to cultivate.

The significant negative coefficient for BMI (β = −0.08, *p* = 0.014) in the linear regression warrants careful interpretation. When body image disturbance and sociocultural factors are held constant, higher BMI was associated with marginally lower EAT-26 scores. This should not be read as evidence that higher BMI is in any sense protective against disordered eating. Rather, the follow-up analyses reported in Section 3.3.1 demonstrate a classic suppression pattern [43]: BMI was positively associated with disordered eating attitudes at the bivariate level, this association was carried by the strong link between BMI and body image disturbance, and the BMI coefficient reversed sign once the BAT was controlled. At equivalent levels of body image disturbance and sociocultural pressure, higher BMI per se does not independently elevate disordered eating cognitions. The observed bivariate relationship between BMI category and disordered eating (the chi-square finding) is better understood as mediated through body image disturbance—it is the BAT that carries the explanatory weight, not BMI directly.

### 4.4. Clinical and Public Health Implications

The findings of the present study carry several implications for clinical practice and public health in Hungary. The 20.7% prevalence figure, in a community adult sample with a mean age of 44 years, suggests that disordered eating attitudes are a normative rather than exceptional feature of the Hungarian adult population and are not confined to the demographic groups—young women, high-BMI individuals—most commonly targeted by screening initiatives. The bimodal BMI pattern reinforces the need for psychometric screening that is decoupled from weight status. Clinicians working in primary care, endocrinology, and bariatric medicine contexts are well positioned to administer the EAT-26 as a brief and psychometrically robust screening tool.

The primacy of BAT across all analyses identifies body image disturbance as the most important intervention target in this population. Psychological interventions with an established evidence base for body image—including Cognitive Behavioural Therapy (CBT), Acceptance and Commitment Therapy (ACT), and body image-specific group programmes—are therefore most likely to produce downstream reductions in disordered eating attitudes. Interventions targeting sociocultural pressures (e.g., media literacy programmes, appearance ideal deconstruction) may exert indirect benefit by reducing the inputs that generate body image disturbance, but the present findings suggest they would have limited direct impact on eating attitudes once body image is already disturbed. The finding that SATAQ-3 Athletic internalisation retains a direct predictive path suggests that sport and fitness contexts—including gym culture, competitive athletics, and fitness-oriented social media consumption—warrant targeted attention in prevention efforts, particularly given the cross-gender nature of this effect.

These findings also carry implications for digitally informed prevention. Social media literacy programmes, which extend classical media literacy training to the interactive and algorithmic features of contemporary platforms, have shown promise in reducing appearance-ideal internalisation and body dissatisfaction [45]. The direct pathway from athletic internalisation suggests that prevention efforts should explicitly address fitspiration content and fitness-oriented platform use—in men as well as women—and that clinicians and dietitians should routinely enquire about appearance-focused social media consumption when screening for disordered eating. Given the volume of diet-related misinformation circulating online [8], nutrition professionals are also well placed to counter restrictive and pseudoscientific dietary content with evidence-based guidance.

With respect to instrument selection for adult screening in primary care, the present findings support the EAT-26 as the instrument of choice: it is brief, was psychometrically robust in the present adult sample (α = 0.84), carries an established clinical cut-off, and detected clinically meaningful attitudes across the full adult age range. Two caveats apply. First, screening classification is threshold-dependent: as the logistic analyses demonstrated, sensitivity in community samples with unbalanced base rates improves markedly when classification thresholds are calibrated to the base rate rather than fixed at conventional defaults. Second, no simple anthropometric index can substitute for psychometric screening. Alternative body shape indices such as A Body Shape Index (ABSI) [46] or the Body Roundness Index (BRI) [47] improve on BMI as descriptors of adiposity distribution and associated somatic risk, but—like BMI—they index body composition rather than eating-related cognition. Given the present finding that the BMI–disordered eating association is carried by body image disturbance, and that elevated rates occurred at both extremes of the weight spectrum, anthropometric measures of any kind are better viewed as complements to, rather than substitutes for, brief psychometric screening.

**Figure 1:**
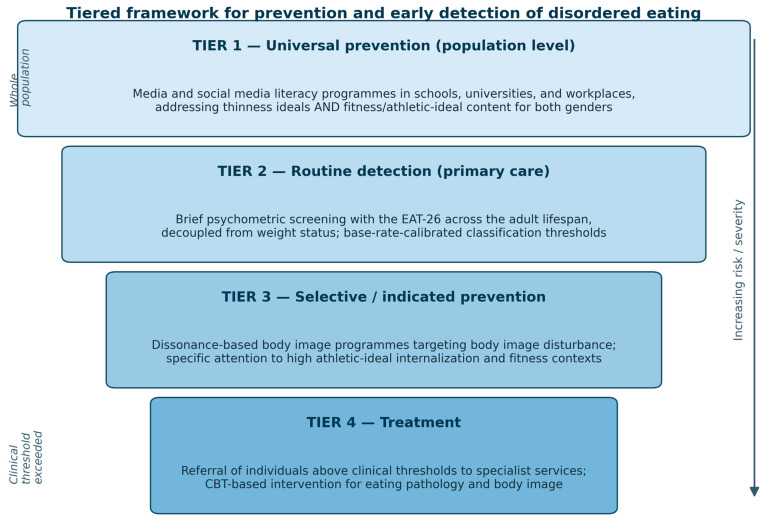
A proposed tiered framework for the prevention and early detection of disordered eating in the Hungarian healthcare context, derived from the present findings.

Finally, the present findings can be organised into a tiered framework for the prevention and early detection of disordered eating in the Hungarian healthcare context (Figure 1). At the universal level, media and social media literacy programmes in schools, universities, and workplaces address the sociocultural inputs identified here, with content explicitly covering fitness- and athletic-ideal material in addition to thinness ideals [45]. At the level of routine detection, brief psychometric screening with the EAT-26 in primary care—decoupled from weight status and applied across the adult lifespan—follows directly from the prevalence and BMI findings. At the selective/indicated level, evidence-based body image interventions, most notably dissonance-based prevention programmes [48], target the proximal correlate identified in all analyses—body image disturbance—with fitness-oriented populations warranting specific attention given the athletic-ideal pathway. At the treatment level, individuals exceeding clinical thresholds require referral to specialist services offering CBT-based intervention for eating pathology [16]. This framework is a proposal derived from cross-sectional evidence; its implementation and evaluation in the Hungarian healthcare system remain tasks for future research.

### 4.5. Limitations

Several limitations of the present study should be acknowledged. First, the cross-sectional design precludes causal inference: while the regression models are framed in predictive terms, the directionality of the relationships between BAT, SATAQ-3, and EAT-26 cannot be established from these data. Second, BMI was calculated from self-reported height and weight, which is subject to systematic error—most commonly underreporting of weight and overreporting of height—potentially attenuating the observed associations involving BMI [49]. Third, the sample was recruited via an online platform, introducing self-selection bias; individuals with heightened interest in or concern about eating, weight, and appearance may be overrepresented relative to the general Hungarian adult population, potentially inflating prevalence estimates. Relatedly, women constituted 70.8% of the sample. Although gender-disaggregated statistics are reported throughout and men and women did not differ in age or BMI, the overall prevalence estimate is weighted toward female response patterns and should not be generalised to the Hungarian adult population without adjustment; population-weighted replication would strengthen the prevalence conclusions. Similarly, while the broad age range (16–81 years) is a strength for lifespan coverage, it introduces developmental and cohort heterogeneity; the age analyses reported here address this in part, but age-stratified replication in larger samples is warranted. Fourth, the logistic regression model demonstrated markedly lower sensitivity for the disordered eating group (41.4% correctly classified) than for the non-disordered eating group (94.6%), reflecting a high false-negative rate that would have significant consequences in a clinical screening context. The threshold analyses reported in Section 3.3.2 indicate that this reflects the interaction of the default 0.50 classification threshold with the 20.7% base rate rather than poor discrimination per se (AUC = 0.83); nevertheless, any applied use of such a model for screening would require prospective calibration and validation. Fifth, gender was operationalised as a binary variable in the present dataset; the experiences of non-binary and gender-diverse individuals were not captured, limiting the generalisability of the gender comparison findings. Finally, no clinical interview was conducted to confirm eating disorder diagnoses; the EATCategory variable reflects screening-level classification, not clinical diagnosis. In addition, the study did not include a direct measure of social media use, platform-specific exposure, or engagement with appearance-focused online content. The SATAQ-3, although its Information and Pressure subscales index media-transmitted influence, was developed prior to the emergence of social media and does not capture platform-specific processes such as algorithmic curation, online appearance comparison, or fitspiration exposure. The associations reported here may therefore underestimate the contribution of contemporary digital appearance pressures to disordered eating attitudes. For this reason, all references to social media, fitspiration, and algorithmic amplification in this article should be read as theoretical context drawn from the wider literature rather than as conclusions licenced by the present data; the constructs actually measured are awareness of, perceived pressure from, and internalisation of media-communicated appearance ideals as operationalised by the SATAQ-3. Successor instruments (SATAQ-4, SATAQ-4R) [24,25] were designed for the contemporary media landscape and should be preferred in future Hungarian research once validated adaptations become available.

### 4.6. Future Directions

Future research should address several of the gaps identified by the present study. The mediation analyses reported here were necessarily cross-sectional; structural equation modelling with latent variables would additionally allow the full mediational system to be estimated simultaneously while accounting for measurement error. Longitudinal designs would further enable examination of the temporal precedence and directionality of these relationships, a necessary step for establishing causal models of eating disorder risk. Gender-stratified regression models would allow for investigation of whether the predictive architecture differs between men and women—in particular, whether SATAQ-3 Athletic operates more strongly for one gender in the context of the other predictors. The inclusion of male-specific body image and eating pathology measures, such as the Drive for Muscularity Scale [50] or the Muscle Dysmorphic Disorder Inventory [51], would enrich the theoretical model for male participants. Replication of the present model in a clinical Hungarian sample would allow for cross-validation of the predictor weights and examination of whether the dominance of BAT is maintained at higher levels of eating pathology severity. Future studies should also incorporate instruments developed for the current media landscape—notably the SATAQ-4 and SATAQ-4R [24,25]—alongside direct measures of social media use, appearance-focused engagement, and fitspiration exposure. Ecological momentary assessment designs would permit fine-grained. examination of how everyday digital exposure translates into state body dissatisfaction and eating cognitions, and would allow the athletic internalisation pathway identified here to be traced to its putative digital sources.

## 5. Conclusions

The present study provides the first integrated examination of disordered eating attitudes and their psychological and sociocultural predictors in a Hungarian adult community sample. The finding that approximately one in five Hungarian adults scored above the EAT-26 clinical threshold highlights the public health relevance of disordered eating beyond clinical settings. Body image disturbance, as measured by the BAT, emerged as the dominant predictor of disordered eating attitudes across all analyses, and formal mediation analyses confirmed that the associations of sociocultural appearance pressures with eating attitudes were carried largely through body image disturbance, consistent with cognitive-behavioural models that position negative body evaluation at the core of eating pathology. Gender differences were robust across most constructs, with the notable exception of SATAQ-3 Athletic internalisation, which showed equivalent endorsement in men and women and retained a direct predictive path to disordered eating independent of body image disturbance. This finding points to the growing cross-gender relevance of athletic body ideals in contemporary culture and their specific association with eating pathology, while the cross-sectional design and the absence of direct social media measures mean that digital-media interpretations of this pathway remain to be tested directly. Collectively, these findings have direct implications for screening, prevention, and intervention efforts targeting disordered eating in Hungary and contribute empirical evidence to the cross-cultural generalisability of established eating disorder models.

## Figures and Tables

**Table 1:** Descriptive Statistics for Anthropometric Variables, Overall and by Gender.

Variable	Total *M* (*SD*)	Male *M* (*SD*)	Female *M* (*SD*)	*t*	*p*
Age (years)	44.09 (13.69)	44.84 (13.04)	43.78 (13.95)	0.92	0.360
Height (cm)	169.83 (9.11)	179.27 (7.54)	165.94 (6.46)	23.12	<0.001
Body weight (kg)	77.32 (18.65)	87.08 (15.38)	73.30 (18.40)	9.26	<0.001
BMI (kg/m^2^)	26.75 (6.04)	27.12 (4.91)	26.60 (6.45)	1.14 †	0.255

*Note. N* = 669–675 (male 196–197, female 472–478); cell values are *M* (*SD*). *t* and *p* values are for the independent-samples comparison of men and women. † Welch’s test (unequal variances), *df* = 473.08; all other comparisons used *df* = 667–673. Men and women did not differ significantly in age or BMI. *Note.* BMI = body mass index, calculated as weight in kilograms divided by height in metres squared.

**Table 2:** BMI Category by Disordered Eating Group: Frequencies and Row Percentages.

BMI Category	No Disordered Eating	Disordered Eating	Total
Underweight	14 (70.0%)	6 (30.0%)	20 (3.0%)
Healthy Weight	226 (81.6%)	51 (18.4%)	277 (41.3%)
Overweight	177 (83.5%)	35 (16.5%)	212 (31.6%)
Obese	116 (71.6%)	46 (28.4%)	162 (24.1%)
Total	533 (79.4%)	138 (20.6%)	671 (100.0%)

*Note.* Percentages reflect the proportion within each BMI category. Disordered eating = EAT-26 score ≥ 20.

**Table 3:** Independent Samples *t*-Test Comparing Male and Female Participants.

Variable	Male M (SD)	Female M (SD)	t	*df*	*p*	Cohen’s d
SATAQ-3 Information	19.21 (7.48)	21.39 (7.87)	−3.39	382.67	<0.001	−0.28
SATAQ-3 Pressure	13.88 (6.36)	16.44 (7.28)	−4.54	415.14	<0.001	−0.36
SATAQ-3 Internalisation	17.05 (7.22)	20.39 (9.15)	−5.03	458.51	<0.001	−0.39
SATAQ-3 Athletic	12.78 (4.95)	12.86 (5.21)	−0.19	382.56	0.847	−0.02
BAT	25.16 (13.31)	40.07 (20.49)	−11.18	550.44	<0.001	−0.80
EAT-26	8.24 (7.72)	13.69 (9.73)	−7.71	456.66	<0.001	−0.59

*Note.* Welch’s corrected degrees of freedom reported for variables with unequal variances (SATAQ-3 Pressure, SATAQ-3 Internalisation, BAT, EAT-26). BAT = Body Attitude Test; SATAQ-3 = Sociocultural Attitudes Towards Appearance Questionnaire–3; EAT-26 = Eating Attitudes Test–26.

**Table 4:** Multiple Linear Regression Predicting EAT-26 Scores.

Predictor	B	SE B	β	t	*p*
Constant	3.16	1.61	—	1.97	0.049
BMI	−0.13	0.05	−0.08	−2.46	0.014
BAT	0.29	0.02	0.61	15.54	<0.001
SATAQ-3 Information	−0.07	0.05	−0.06	−1.48	0.140
SATAQ-3 Pressure	0.07	0.06	0.05	1.06	0.289
SATAQ-3 Internalisation	0.03	0.06	0.03	0.46	0.646
SATAQ-3 Athletic	0.15	0.07	0.08	2.09	0.037

*Note.* R^2^ = 0.405; Adjusted R^2^ = 0.400; F(6, 664) = 75.40, *p* < 0.001. β = standardised regression coefficient. BAT = Body Attitude Test; SATAQ-3 = Sociocultural Attitudes Towards Appearance Questionnaire–3; BMI = body mass index.

**Table 5:** Binary Logistic Regression Predicting Disordered Eating Group Membership.

Predictor	B	SE	Wald	*p*	Exp(B)	95% CI
BAT	0.06	0.01	90.86	<0.001	1.07	[1.05, 1.08]
SATAQ-3 Information	−0.02	0.02	1.04	0.308	0.98	[0.94, 1.02]
SATAQ-3 Pressure	−0.02	0.03	0.59	0.444	0.98	[0.93, 1.03]
SATAQ-3 Internalisation	0.01	0.03	0.25	0.616	1.01	[0.96, 1.06]
SATAQ-3 Athletic	0.07	0.03	5.50	0.019	1.07	[1.01, 1.13]
Constant	−4.38	0.44	98.51	<0.001	0.01	—

*Note.* Nagelkerke R^2^ = 0.352; χ^2^(5) = 172.05, *p* < 0.001. Overall classification accuracy = 83.6%. Exp(B) = odds ratio. BAT = Body Attitude Test; SATAQ-3 = Sociocultural Attitudes Towards Appearance Questionnaire–3.

**Table 6:** Mediation of the Associations Between SATAQ-3 Subscales and EAT-26 Scores Through Body Image Disturbance (BAT), Controlling for BMI.

Predictor (X)	*a*	*b*	*c*	*c*′	*ab* [95% CI]
Information	0.63 ***	0.32 ***	0.21 ***	0.01	0.20 [0.14, 0.27]
Pressure	1.08 ***	0.30 ***	0.41 ***	0.08	0.33 [0.25, 0.41]
Internalisation–General	1.11 ***	0.30 ***	0.41 ***	0.07	0.33 [0.27, 0.40]
Internalisation–Athletic	1.39 ***	0.30 ***	0.59 ***	0.17 **	0.42 [0.32, 0.52]

*Note. n* = 671. Unstandardised coefficients. *a* = X → M path; *b* = M → Y path (controlling X); *c* = total effect of X on Y; *c*′ = direct effect of X on Y (controlling M); *ab* = bootstrapped indirect effect with 95% percentile confidence interval (5000 resamples). All models control for BMI. ** *p* < 0.01; *** *p* < 0.001.

## Data Availability

The data presented in this study are available from the corresponding author upon reasonable request. The data are not publicly available due to privacy, confidentiality, and ethical restrictions related to the protection of participants’ personal information.
